# Death, unity, and the brain

**DOI:** 10.1007/s11017-019-09479-8

**Published:** 2019-04-03

**Authors:** David S. Oderberg

**Affiliations:** Department of Philosophy, University of Reading, Reading, RG6 6AA UK

**Keywords:** Death, Brain death, Organ donation, Bodily integration, Hylemorphism

## Abstract

The dead donor rule holds that removing organs from living human beings without their consent is wrongful killing. The rule still prevails in most countries, and I assume it without argument in order to pose the question: is it possible to have a metaphysically correct, clinically relevant analysis of human death that makes organ donation ethically permissible? I argue that the two dominant criteria of death—brain death and circulatory death—are both empirically and metaphysically inadequate as definitions of human death and therefore hold no epistemic value in themselves. I first set out a neo-Aristotelian theory of death as separation of soul (understood as organising principle) and body, which is then fleshed out as loss of organismic integrity. The brain and circulatory criteria are shown to have severe weaknesses as physiological manifestations of loss of integrity. Given the mismatch between what death is, metaphysically speaking, and the dominant criteria accepted by clinicians and philosophers, it turns out that only actual bodily decomposition is a sure sign of death. In this I differ from Alan Shewmon, whose important work I discuss in detail.

## Introduction

Is it possible to have a metaphysically correct, clinically relevant analysis of human death that makes organ donation possible? The enormous difficulty of finding an answer to this question is matched only by the grave ethical implications any answer will have. Considering that the first human organ transplant was performed in 1954 and that in 2015 over 126,000 were performed worldwide [1], there can be no surprise at the exertions made by clinicians and many bioethicists to secure as many organs as possible so as to give life and health to as many people as possible.

Of the various ethical problems to which transplantation gives rise, the problem of death is in my view the most pressing. The dead donor rule, as it is called, still prevails in most countries: vital organs must only be removed from dead patients [2]. In other words, removing vital organs must not itself *cause* the death of the patient [3, p. 297].^1^ The problem, though, is that ‘the tension between the need for both “live organs” and a “dead donor” has required the development of very explicit criteria for declaring the “moment” of death, despite the absence of a biological basis for this degree of precision’ [4, p. 106]. The two most common criteria of death are ‘whole brain death’ and ‘circulatory death’, both enshrined in the 1980 Uniform Determination of Death Act [5]. Yet there is a welter of disagreement among clinicians and ethicists as to which, if any, criterion is correct and why. Whole brain death—consisting in the “irreversible cessation of all functions of the entire brain, including the brain stem” [5, §1]^2^—still holds sway as the favoured standard for determining the death of the human being.^3^ It has, however, come under persistent pressure from its critics, most notably Alan Shewmon (e.g., [11–13]) and Robert Truog [14, ch. 3]. Some of its defenders, perhaps surprisingly, consider themselves Thomists and hold that the test is consistent with the Scholastic metaphysics exemplified by Thomas Aquinas [15, 16]. Other Thomists have rejected this attempted reconciliation of Thomistic metaphysics with the whole brain criterion [17].

My purpose here is not to take on the large task of evaluating the whole brain criterion in all its aspects, let alone to investigate the various other criteria that have been proposed. Rather, I examine some important issues surrounding the whole brain and related criteria. The debate is currently sagging both under the weight of empirical and diagnostic information appealed to by partisans of the various sides, arguments, and counter-arguments and under what seems to me a general anxiety over the lack of consensus concerning what death is, how it is determined, and whether human beings even need, ethically, to *be* dead before their organs are removed.

It is not possible to approach these questions all together, nor is it desirable to do so. I have to make certain assumptions, which may limit the appeal of what I argue. I assume without argument that the dead donor rule is ethically correct: removing (vital) organs from living human beings—at least without their consent^4^—is not merely as good as wrongfully killing them; it *is* wrongfully killing them. I make no distinction between the human person and the human being, as some do [18]. I further assume a broadly Aristotelian metaphysic of the human being. That is, the human being is a compound of form and matter, where form is, to put it as neutrally as possible, the ‘organising principle’ that unifies matter into a functioning, integrated whole. There is no need to assume that the human form—which Aristotelians call the soul (*psuchê*)—survives a human’s death. Rather, I take such an assumption to be irrelevant for present purposes. The only metaphysical loading, as it were, that is given to the term *soul* in the present analysis is that of organising or unifying principle. I also understand the human being as an essentially rational animal—a being for whom the power of rational thought is present as long as it is a normal member of its kind, just as being winged is essential to birds as long as they are normal members of their kind.^5^ Again, however, the present discussion does not rely on this understanding.

I have suggested that these assumptions may limit the value of the following discussion, but that would depend on how much specificity is packed into the assumptions. If one thinks that they cannot even be rendered plausible without taking on a raft of contentious ethics or metaphysics, then the discussion may indeed have limited appeal. I am not so sure. Stripped of any tendentious terminology, one does not have to be an Aristotelian or an unqualified defender of the sanctity of life to find merit in the following ideas: killing people for their organs is wrong; human beings and human people are one and the same; humans are distinctively rational beings; what makes an organism a living member of its kind is its functional unity and integrity. Taken in this relatively ‘thin’ way, the assumptions—and the analysis I offer using them as my starting point—are worthy of consideration even if the reader does not share a particularly Aristotelian way of understanding the world. That said, I frame my case in an unapologetically Aristotelian-Thomistic fashion, in the hope that the reader will find some merit in the more detailed metaphysical framework I adopt (along with not a few other writers in the debate over death).

With this background in place, I proceed as follows. First, I set out the proper Aristotelian understanding of death as soul–body separation. This understanding is then applied to the idea of death as the loss of integrity. Next comes a consideration of the popular view that the brain is the integrator of the human being, the idea being that if the brain ceases to function, then the organism ipso facto loses integrity and is therefore dead. I then examine the alternative theory that ‘circulatory death’ is the death of the human being. Next, I look at important questions concerning irreversibility: is it necessary for death, and if so, can irreversibility ever be verified? My conclusions will not be of much assistance to advocates of organ transplantation who accept the dead donor rule, given the sure criterion of death that I end up proposing—but this is not my concern. More encouragingly, my proposal does not preclude the future technical possibility of respecting the dead donor rule while maintaining a supply of usable organs.

## Death as separation

Death, put simply, is the separation of the soul from the body. This does not imply either that the soul continues to exist after death or that it does not. As far as the present discussion goes, it implies only that death is the loss of the body’s organising principle. A clay statue ceases to exist when it loses its shape, but the shape does not continue to exist. Nor does the idea of death as separation imply that the body continues to exist after death. Following Aristotle, the post-mortem body is the body of a dead person in name only. In reality, the body has been replaced by a lump of flesh on the way to carrion. When a statue is smashed to bits, its form is separated from its matter, but neither the form nor the lump of clay continues to exist.^6^ True, we do not typically say that a statue’s lump of clay has been separated from its shape, but this is what the loss amounts to. Now, a statue is not a substance but an artefact, so its form is not substantial but accidental (albeit essential—part of its essence is to have a certain accident, in the Aristotelian sense).^7^ And its form is just its shape. Neither of these facts is true of humans, but the general point applies: a form and its matter can be separated without the form’s surviving. When a non-human animal dies, it too suffers the separation of form and matter, but there is no reason therein for thinking that its form survives.

The death of the human being consists, then, in nothing other than the loss of his form; to die is, simply, to lose your soul—understood as your metaphysical principle of organisation or unity. Is this loss or separation gradual or instantaneous?^8^ The answer is that it depends, since *loss* or *separation* can mean either (i) the process of losing or separating or (ii) the coming to be of the result of this process—its *culmination*. The process, by definition, has to be gradual. Being gradual does not entail being slow (relative to some standard) or prolonged, but simply taking place over a temporal interval. Hence most if not all of the cases called *sudden death*—say, by electric shock or powerful trauma—are (or at least could be) absolutely gradual yet relatively sudden by comparison to, for example, drowning or poisoning.

Separation as the *culmination* of the process—the event of *becoming dead*, strictly speaking—has, however, to be instantaneous. Even without an underlying hylemorphic ontology, whereby the organism is a form-matter compound, it is hard to see how life and death can be other than all or nothing.^9^ What could it mean to be partially dead? An organism might be *partly* dead, say, by having necrotic tissue or a nerveless tooth; but an organism that is partially dead would have to engage simultaneously in the behaviour of a living thing and the behaviour of a dead thing, and if being dead means not existing, the organism would have to behave in ways consistent both with its existing and not existing—which is absurd. If life and death are all or nothing, therefore, the event of *becoming dead*—the culmination of the process of dying—cannot obtain via a series of stages in which an organism starts off alive and then undergoes stages of partial death until it is wholly dead. Yet for the event of becoming dead not to be instantaneous (i.e., to be gradual), the organism would have to pass through such a series of stages. Otherwise, how else could the outcome be achieved gradually?

No counterexample to my claim is to be found in the case of a body gradually consumed by, say, gangrene or some toxin that progressively destroys the tissue. This would be a clear case of gradual dying—separation in sense (i) above—but not of gradual death—separation in sense (ii). One should agree with Shewmon’s endorsement [13, p. 429] of James Bernat and colleagues’ distinction between the ‘whole organism’ and the ‘organism as a whole’ [10, p. 390]. An organism can lose parts, or it can endure the disintegration of function or components in a localised region, and so not be a whole organism according to the standards of the kind to which it belongs—standards including material constitution and function both at a time and over time. Yet it can still retain its status as an organism—a being that, overall or as a whole, given the relevant standards, maintains the essential unity of constitution and function without which it would otherwise cease to exist altogether. An organism slowly consumed by gangrene is every bit as alive as a healthy one, the difference consisting in how close to death each one is. Dead parts do not of themselves render an organism dead. Moreover, this is just what should be expected from a hylemorphic understanding of life. An organism’s matter is informed: it is organised, structured, and determined as an entity of a specific kind with a characteristic repertoire of behaviour and operation. Given that what does the organising is a real metaphysical principle—a universal form particularised in some matter—it is hard to see how the form could be only partly present. How could it inform some but not all of the organism? Moreover, informing all of the organism means informing *every single part* of the organism down to the smallest sub-atomic parts. If it did not do so, then we would have to say that the putative parts that were not informed by the organismic form were not parts of the organism at all. Gut or skin bacteria, for instance, no matter how strong their symbiotic relationship to the host symbiont organism, are not literal parts of that organism because they are not informed by the organism’s own form. Moreover, denying the all-or-nothing nature of the organism’s informance^10^ by its form is almost certainly inconsistent with the doctrine of the unicity of substantial form—the distinctively Thomistic doctrine, based on good Aristotelian reasons, that there is one and only one substantial form, or more loosely organising principle, for each individual substance.^11^ For the obvious (at least to an Aristotelian) way of maintaining that an organism can be only partially informed by its organismic substantial form would be to hold that some part of that organism was informed only by a vegetative or sensitive form but not by the higher form proper to the organism. To put it non-Scholastically, the idea is that in a human, say, some parts are merely vegetative or sensitive (merely animal) but not fully human, while other parts are fully human. This might be possible for some kind of freakish, Frankensteinian creation, but I have no idea how it could apply to a real, unitary organism such as a human being. To what vegetative or animal kind would these ‘partially human’ parts belong, biologically speaking? Their DNA would be human, and mere damage or malfunction would not be sufficient to deprive them of their human identity. I will not spend any longer on this rather fanciful way of defending partial informance, leaving the defender of a plurality of forms to make sense of it.

So, if one takes together: the idea of death as separation; the notion that separation is an event distinct from the process leading up to it; the view that hylemorphic composition—at least in the case of organisms—is all or nothing; and the recognition that one clear way of denying it requires also denying the doctrine of unicity; there is, then, a strong case for regarding death as instantaneous.^12^ It is not for nothing that the expression ‘the moment of death’ has a long history. Hence one should not misinterpret the Jesuit moral theologian Juan Ferreres, who in his important early twentieth-century work says: ‘in general we may hold as universally admitted that death does not invade the entire organism suddenly, but only gradually, the separation of soul and body taking place some time after the man is usually said to be dead’ [21, p. 58]. Immediately following, he adds: ‘That there exists a longer or shorter period of life between the actual moment of death and that ordinarily supposed to be indicated as such by certain symptoms is generally admitted’ [21, pp. 58–59]. He then refers to Jean Laborde, whose significant early work on resuscitation occupies a large part of Ferreres’ book; Laborde speaks of ‘the moment at which the spark of life is totally and finally extinct’ [21, p. 59].

## Death as disintegration

There is, however, an immediate challenge to the idea of the moment of death as a genuine *moment*. Most, it seems, of the dead donor rule’s defenders stress the centrality of integrity to organismic life. Whether they support the whole brain death criterion or not, they agree that the loss of integrity of the organism is the biological fact in which death consists. The disagreement, of course, is over whether that fact should be equated with the fact of whole brain death or some other fact.^13^

The challenge is that integrity—or integration, which is the more common term—looks like it is not an all-or-nothing matter, meaning that loss of integrity must be gradual and so cannot be instantaneous. This appearance is lent support by the ways in which supporters of integrity formulate the phenomenon. Shewmon, for example, speaks of integrity as ‘the anti-entropic mutual interaction of all the cells and tissues of the body, mediated in mammals by circulating oxygenated blood’ [11, p. 473]. Nicholas Tonti-Filippini speaks of ‘intercommunication between the parts in such a way that the body remain[s] a functional whole’ and says that ‘the parts of the whole are intercommunicative with each other as a dynamic unity,’ something which, he notes, can be ‘partial’ [22, pp. 321, 318, 319]. Again, Bernat et al. hold that ‘the spontaneous and innate interrelationship of all or most of the remaining subsystems and the interaction of the perhaps impaired organism with its environment is to be regarded as the functioning of the organism as a whole,’ where functioning is equated with the integrity of the organism [10, p. 390].

I could multiply instances but the point is clear enough. If integrity is understood in terms of intercommunication or transfer of information between parts and subsystems, the interaction of the organism with its environment, and the like, then it is hard to see how integrity has to be all or nothing. Not all parts need receive all biologically normal kinds of communication—for example, a paralysed limb will not receive nerve impulses, and nerve signals might be present in some parts where circulation is severely compromised. Not only can integration gradually be lost, it might be thought, but there can also be no moment marking the leap from life to death since there is no moment marking the leap from integration to non-integration. Proponents of integration as communication do not, as far as I can tell, identify the moment of death as the leap from the cessation of the final organismic system or information transfer process left functioning to the absence of all such systems or processes. Even if proponents of this view made such an identification, the fact that the last system—whatever it might be—does not itself shut down instantaneously would still make the transition from life to death non-instantaneous, on this view.^14^

Given this mismatch between loss of integration as a biological phenomenon and separation as a metaphysical one, it is exceedingly difficult to see how the two could be identified. Might it be enough that they were co-extensive, perhaps necessarily so? In this case, would integration not be an acceptable epistemic criterion or test of separation? The problem, though, is that there is no reason to think that any particular kind or degree of loss of integration, on the intercommunication model, is *the* phenomenon whose co-extensiveness with separation is required here. And that is another reason for not identifying such loss with separation of body and soul. Just what *is* the biological phenomenon in question? When one looks for communication and transfer of information between parts and sub-systems, why are such phenomena considered metaphysically important?

The answer, according to the hylemorphic theory, is that these phenomena are marks of integrity in the all-or-nothing sense. The idea of ‘the organism as a whole’, of course, gestures in just the right direction, so it is not as though integration theorists are missing the point. But they should not focus on intercommunication between parts, which is a matter of degree and comes in many kinds. Otherwise, there is no obstacle in the way of holding that declining integration is just gradual death (rather than gradual dying) and that if enough, but not all, systems shut down or stop interacting, the human being is not fully alive, or is ‘as good as dead’—perhaps good enough for organs to be removed.

Integrity is all or nothing.^15^ Either the organism is a whole or it is not; the concept of a *partial whole*, at least for organisms, makes no sense. When the body is informed, integrity is present and there is a whole organism; when form is lost, so is integrity and no organism remains at all. This is how form works—it unifies matter into a substantial whole and permeates every part of the substance; my big toe is as much me (as long as it is attached and functioning) as my head or, for that matter, my brain. The same goes for an individual red blood cell or a single neuron. Form reaches into every part of the organism; if it did not do so, an organism would be an accidental unity—a complex of at least two substances or of a substance and at least one accident. An organism as a whole remains so even when it is less than a whole organism.^16^ Loss of integration, in the sense of intercommunication, should make no more difference to integrity in the all-or-nothing, hylemorphic sense than should amputation.

Given this metaphysical approach to death, the concept of death as disintegration needs to be handled with extreme care. For one must not confuse the all-or-nothing, instantaneous loss of integrity in which death consists with one or other process whereby the parts and subsystems of an organism cease, perhaps gradually, to function one by one. Moreover, there is a third concept of disintegration which must be kept separate—disintegration as the literal physical separation of parts of an organism. It is my contention that disintegration as systemic failure—the second concept—can only occur prior to disintegration as loss of integrity, which is true metaphysical death. Further, disintegration as physical separation can occur either prior or subsequently to true metaphysical death. Epistemically, systemic failure is, of course, a fallible sign of dying, whether or not the organism in fact dies. Physical separation is also a fallible sign of dying when it occurs prior to death. And when it occurs after death, it is a sign—this time infallible—that the organism has died. The third concept of disintegration—physical separation—plays a far more important role in the debate over organ donation than so far acknowledged by any side. I return to this towards the end, but first I need to consider the challenge that the brain death criterion poses to the approach I have outlined, followed by the role of the circulatory test as, perhaps, more congenial to the hylemorphic theory.

## Is the brain the integrator? Empirical objections

Within the long-running, overall debate as to whether brain death is death of the human lies the sub-debate as to whether the brain is the integrator of the human organism. Defenders of the brain death criterion who agree that death is a loss of bodily integrity argue that said integrity is lost when the whole brain ceases to function, precisely because the brain *is* the integrator of the organism [6, p. 35; 10; 16; 22; 23]. As one defender of brain integration puts it, ‘without the brain, the body loses its form, so to speak, as the parts cease to be an integrated dynamic unity’ [22, p. 313]. Or, in less high-flown terms and without the semi-Aristotelianism:

A moment’s reflection discloses that it is primarily the brain that is responsible for the functioning of the organism as a whole: the integration of organ and tissue subsystems by neural and neuroendocrine control of temperature, fluids and electrolytes, nutrition, breathing, circulation, appropriate responses to danger, among others. The cardiac arrest patient with whole brain destruction is simply a preparation of unintegrated individual subsystems, since the organism as a whole has ceased functioning. [24, p. 48]^17^

Note that I am not concerned here with the distinct position that brain death (whether whole or part) is human death because the brain is the ‘seat of consciousness’ or ‘locus of personhood’, or a similar notion.^18^ I find such views highly implausible, and they would in any case require separate evaluation. Rather, my concern is with the popular idea that the functioning brain is a kind of ‘master part’ that integrates the body and so is a necessary condition of the very existence of the human being.

The integrator theory, for all its prima facie plausibility, faces enormous problems apart from the general ones affecting all whole brain theories—such as the virtual impossibility of determining that the entire brain has ceased functioning. First, it is necessary to know whether the integrator theory is an empirical or metaphysical theory. Is it that physiology teaches that the brain is the integrator of the organism, or is it that one comes to this as a *metaphysical* view from empirical facts about the brain’s workings in respect of the organism? Neither interpretation is palatable.

Taken as an empirical theory—say, as the systematisation of a large number of pertinent empirical facts—the position has to be something like the following: the brain controls so many organism-level systems, processes, and states that when it ceases to do so, one simply cannot speak of there being an organism present at all. Specifically, ‘the intrinsically vital functions provided by the heart and other organs are wholly dependent on the cohesive and regulatory functions of the brain’ [25, p. 37] (quoted in [11, p. 463]). The problem with this view is that it has been thoroughly refuted on empirical grounds, as the extensive work of Shewmon has demonstrated.^19^ As Franklin Miller and Robert Truog put it, ‘Shewmon has described how most of the brain’s functions are not directed toward integration of the organism, and how most of the integrative functions of the body do not require brain function’ [14, p. 65].^20^ For example, a variety of homeostatic processes take place in patients diagnosed as brain dead, along with metabolism and catabolism, wound healing, immune responses, bodily growth, sexual maturation, and even gestation to term of a child inside a pregnant, brain-dead woman. Needless to say, not all of these processes will be found in all patients diagnosed as brain dead, though I suspect that not a single patient so diagnosed, by any existing criterion, has failed to display at least one such characteristic.

There are many points that could be raised about these phenomena, but I want to highlight some of the most important. One is that it is always open to a critic to make the ‘no true Scotsman’ move against Shewmon: none of the patients in whom organism-level activity can be found is truly brain dead. It is not as though such a move would in itself be invalid if there were a definitive test that such patients actually failed to meet. And the information I am appealing to here derives from cases where, by and large, brain death was formally declared based on widely agreed upon criteria. Nevertheless, the criteria are not identical in every case, and the fact is that there is no single ‘gold standard’ test of brain death, which is a major concern. Perhaps clinicians will, one day, rally around one clear criterion of brain death—but this merely places the onus on defenders of brain death to make their case. Until then, opponents can only rely on the tests—many of them extremely rigorous—already in widespread use. This does not, however, allay the main worry—that is, whether any particular empirical fact is *the* fact of brain death. Must every last neuron have ceased to function? Must no single neurotransmitter be working? There is nothing frivolous about the concern, as long as one thinks that there is a gulf between being ‘as good as dead’ and being actually dead. In one extreme case (of many that could be cited), a boy diagnosed as brain dead from meningitis at the age of four lived another twenty years at home on a ventilator. Autopsy revealed brain destruction with, as Miller and Truog put it, ‘an entirely calcified brain, with no neural elements visible either grossly or microscopically’ [14, p. 64].^21^ Is it that in chronic cases such as these scientists have not yet found *the* neurological component that continues to function in order for bodily integrity to be maintained?

Another point is that there is no conundrum when handling cases of organism-level activity accompanied by brain death. One is not compelled to refine the neurological criterion in order to rule out such cases on *empirical* grounds—as opposed to organ demand-driven grounds. On the contrary, Shewmon offers many reasons for thinking that the survival of a human being with brain death is both possible and predictable, summed up in his theses: ‘(1) most brain-mediated integrative functions are actually not somatically integrating, and (2) most somatically integrative functions are not, in fact, brain-mediated’ [11, p. 463]. To give but one important example, it is well known that patients diagnosed as brain dead need to be mechanically ventilated. It cannot, however, be inferred from this that breathing—as an integrative function—is brain mediated. Moving air in and out of the lungs is indeed brain mediated, but it is not integrative: it is absent in a foetus and bypassed altogether in cases of extracorporeal membrane oxygenation. On the other hand, breathing understood as the entire process of respiration—exchange of oxygen and carbon dioxide—is a whole-organism process that is not brain mediated [11, p. 464].

A criticism of Shewmon’s litany of non-brain-mediated integrative processes is that if any of the putative organism-level processes he appeals to can also be demonstrated in independent cells or cultures outside the body, there is weaker support than might be thought for the idea that organismic integrity obtains despite brain death [23, pp. 258–259]. Clearly the clinical and physiological details are of paramount importance here, and it is true that the process of wound healing, for example, is to some extent demonstrated ex vivo. But it is the extent that makes the difference. Ex vivo models of wound healing require complex preparations for the tissue samples (usually skin) to be viable. One study used incubation in foetal calf serum in order to see any healing at all [28]. As another study points out, lack of blood supply is a major limitation in ex vivo models [29]. The point is that such models will only ever be approximations rather than replications of what goes on when the organism repairs itself. The closer experimenters try to make their approximation, the more they have to mimic or substitute organismic processes, such as circulation and immune support. In other words, by mimicking organismic behaviour in order to have a reliable model, they tacitly admit that in vivo healing is a holistic process attributable to the organism rather than any of its parts. It invokes, as Shewmon puts it, ‘multiple bodily systems distant from the wound’ [11, p. 475].

The same point applies, even more clearly, to phenomena such as sexual maturation, bodily growth, and maternal gestation to term in brain-dead patients. Are these not by necessity organism-level processes? There is no implicit circularity in appealing to them, since I am not speaking of, for example, maturation *of* the organism or gestation *by* the organism. Rather, it is first noted that certain processes can be identified in the body of a brain-dead patient. This observation then leaves an open question as to whether the processes properly belong to an organism or to, as Shewmon colourfully puts it, ‘a mere “collection of organs” in a bag of skin’ [11, p. 473]. The processes of maturation, growth, and gestation all, on any reasonable interpretation of what is going on, require organisms to undergo them. They each require the integrated functioning of numerous organismic systems (e.g., cardio-respiratory, endocrine, nervous, immune, and lymphatic), along with all the homeostatic processes (e.g., temperature and fluid level regulation) that belong to these and other systems and components.^22^

What of the objection from ectogenesis? Gestation to term in artificial wombs, for mammals up to and possibly including humans, is only a matter of time.^23^ Would this not show that gestation to term is an essentially non-organismic process? I do not see how this follows. Just as with wound healing or skin growth in culture, ectogenesis only ever approximates to what a living organism does when it gestates offspring. In vivo, there are continual exchanges between the mother and the child. The offspring makes changes to the mother’s body, immune system, and so on. There is no such exchange in ectogenesis. I have not seen evidence of its occurrence in brain-death gestation cases, but it would be remarkable were it wholly absent, since viable delivery in such cases requires the mother to be somatically prepared, as it were, for the process to take place. An artificial womb no more demonstrates the non-organismic status of natural gestation than does an incubator.

## Is the brain the integrator? Theoretical objections

In addition to the largely empirical objections raised against the brain-as-integrator thesis, there are serious theoretical or conceptual problems. The first, only hinted at but not exploited by Shewmon [11, p. 473], is based on the simple fact that the human organism does not begin its existence with a brain. Neural progenitor cells—the stem cells capable of producing, but not yet actually producing, all the cells of the brain—do not even appear until the third week after fertilisation. The three primary structures—forebrain, midbrain, and hindbrain—are not formed until the fourth week, along with the spinal cord.^24^ I do not intend to defend here the proposition that the human organism exists in the third and fourth weeks after fertilisation; I take it for granted. But if the human organism can and does exist without a brain at some time during its life, how can the brain be the integrator?

There does not seem to be any appealing, non-question-begging way out for the defender of the brain-as-integrator thesis. To say that the brain is the integrator, albeit not essential, will not do. For what could this amount to? If it is a non-essential integrator, then the organism can persist without it, and absent independent reasons for thinking this never happens apart from embryogenesis, the claim either begs the question or proves nothing. There is simply no good reason, given the clear existence of living human organisms without brains, to think that whenever the brain is functioning it is also integrating. On the other hand, the defender might make a modified essentialist claim to the effect that the brain must be the integrator when it is present and functioning. What would be the ground for that? Perhaps it is the idea that once the brain has formed, the organism has reached a certain threshold level of complexity that requires integration by a master organ. Again, however, it is hard to see how this could be more than mere assertion. What level of complexity could constitute this threshold? The natural thought is that it has something to do with differentiation within the organism: the more the cells develop specific functions, the more the organism develops specialised organs and body parts, and the more complex it becomes. Suppose that were true: how would it follow that an integrator was necessary? What reason is there to think that the organism as a whole is incapable of maintaining all of the specialised organs and functions in a cohesive (even if impaired) network of mutually sustaining interactions? In any case, the complexity claim understood in terms of differentiation is false: even by the fourth day after fertilisation, the embryonic blastomeres are differentiating into the inner cell mass and the trophoblasts. If the critic objects that the trophoblasts become extra-embryonic structures and so are not part of intra-embryonic differentiation (a specious point, in my view), consider that by the ninth day—still well before the appearance of neural progenitor cells—the inner cell mass itself differentiates into epiblast and hypoblast.^25^ So differentiation and specialisation start well before brain formation and continue long after. One is, then, still left without a reason for thinking that there is some special level of complexity that requires a master part to take over as integrator in the life of the human organism.

Another serious theoretical objection is briefly mentioned by Michel Accad but not stressed nearly enough [17, p. 222]: what integrates the integrator? After all, the functioning brain itself has the integrity of an organ, so why does it not have its own integrator—some privileged portion of the brain that integrates the whole? The defender of the brain-as-integrator thesis can, of course, deny subscribing to the view that *every* organism, let alone every organ, requires an integrator. He would be right to deny this, since many organisms (and their organs) lack integrators—plants being an obvious example.^26^ But he would still need to give a well-motivated reason for thinking that in the human case, where the organism does, on his view, require an integrator, the latter does not need its own integrator—which is to say, a master part within the organ. After all, if the thought is that the human organism is the sort of thing that needs a master part—whether because of the amount or kind of complexity or because of some other special feature of its operations—it would be hard to deny the same of the brain itself.

The primary problem with requiring an integrator for the brain itself is not that it sets up an infinite regress—something Accad points out [17, p. 222], and a serious problem nonetheless—but that it betrays a misunderstanding of the whole phenomenon of integrity. On the hylemorphic theory, every single part of the organism, down to its very cellular and even chemical constituents, is governed by its form. To repeat my earlier claim, I am as much a human being in my little toe as in my whole body. Everything in the human organism partakes of the human form—and this is not a mere point about DNA. The brain gets whatever functional capacity it has from the very source of all other functional capacity in the organism—the organism’s form. This is demonstrated in the order of efficient causality by the brain’s emergence, as pointed out earlier, from the embryonic developmental process. It is as much a product of the organism’s integral functioning as the hair that ends up growing on the organism’s head. Every part of the organism is the product of a single developmental plan. As such, I submit that it is utterly confused to think that the product of an organism’s integrity could ever be or become the producer of that integrity.

## Circulation—a holistic replacement for the integrator thesis?

I hope I have said enough to show the untenability of the proposition that the brain is the integrator of the organism. As a mainstay of the brain death criterion, the latter is severely weakened thereby. Coupled with my earlier discussion, I have provided strong reasons for thinking that brain death is not the death of the organism. In this I am in full accord with Shewmon and others.

Yet might not the wrongness of the brain death criterion simply invite the search for a criterion of death that is genuinely holistic? If there were an authentic, organism-level process whose cessation entailed the loss of integrity, then one would have such an empirical criterion. It is no surprise that ‘cardio-respiratory’ or ‘circulatory-respiratory’ death, as it is sometimes called, is often appealed to in this regard. (I will henceforth refer simply to ‘circulatory’ death.)

As is well known, circulatory criteria were part of the standard of death in most countries before the advent of brain-based criteria. Traditionally, loss of heartbeat, or pulse, or breathing, have been taken as essential signs of death. Contrary to widespread belief, however, none of these signs, taken together or individually, were ever considered *sufficient* for death.^27^ In the pre-organ donation age, people were far more concerned to ensure that individuals were dead before they were buried or cremated. Loss of sensation, lack of reflexes, absence of spontaneous movement, and more extreme signs, to which I turn shortly, were all part and parcel of the overall determination of death.

Given the challenges to the still-dominant brain death criterion, circulatory death has emerged as a standard of death on its own terms, and as of 2018 12% of United States organ donors were diagnosed as dead by circulatory criteria, up from 2% in 2003.^28^ As with brain death, there is no test of circulatory death that is agreed upon in all details, and patient behaviour varies widely. Once the ventilator is removed, a patient’s heart might continue beating for ten to twenty minutes, and up to several hours [36]. Diagnosis of cardiac death is given around two to five minutes after the heart has stopped beating [39, p. 3]. Rigor mortis and lividity, however, do not begin until at least thirty minutes after the heart stops [40, ch. 1], a point to which I return below.

This variability notwithstanding, one might think that circulatory death is a more promising candidate for the physiological fact that marks the moment of organismic death. Shewmon himself seems to think so.^29^ He considers the full cardio-respiratory-circulatory system—not just the beating heart or pumping lungs, but everything down to the last capillaries—to be genuinely holistic, an irreducibly organism-level property. In contrast to the brain, he asserts, ‘circulation … does reach essentially everywhere, and the few structures without capillaries communicate with the nearest capillaries by diffusion’ [13, p. 465].

Moreover, Shewmon comes very close to endorsing cessation of circulation as the biological sign of the moment of death. He calls it a ‘probably valid criterion’ and an ‘anatomical substrate’ that is ‘close to the moment of death’, specifying the following: ‘cessation of circulation of blood for a sufficient time … to produce irreversible damage to a critical number of organs and tissues throughout the body, so that an irrevocable process of disintegration has begun’ [41, p. 250]. At the same time, Shewmon recognises that ‘there could be many possible valid criteria … that a person has already died. But the closer one tries to get to the unobservable moment of death itself, the more difficult it becomes to formulate a universally valid and certain criterion’ [41, p. 250].

There is a lot to like about what Shewmon says here, but also a serious problem. On the positive side, he adheres clearly to the idea of a metaphysical moment of death that is unobservable. He insists implicitly on organism-level properties or processes as candidates for a criterion of death. And his circulatory criterion is a far better candidate than brain death. Nevertheless, the flaw in his approach lies, first, in his appeal to terms such as ‘irreversible damage’ to critical organs and ‘an irrevocable process of disintegration’, and second, in his claim that there is no single, universally valid sign of death, only more or less approximate criteria that can vary from case to case. Moreover, in another place he makes his position plain: ‘We find it far preferable to say that healthy, living organisms are obviously integrated unities, that decomposing corpses are obviously not unities, and that there is a fuzzy area in between that is intrinsically undecidable’ [42, p. 106].

The first problem concerns irreversibility. There has been a lot of discussion of the term, and Shewmon himself notes the various interpretations that it can be given [42, pp. 95–100]. It figures into most of the widely discussed or agreed upon definitions of death (e.g., [7, 10, 43, 44]). Without entering into a detailed analysis of the different meanings of the term, I claim that any relevant interpretation will be either inadequate or question begging. Suppose *irreversible* means something as weak as ‘permanent’, where permanent cessation of a function (here, cardio-circulatory) means that the function ‘will not restart spontaneously and no medical interventions will be conducted to restart it’ [45, p. 314]. The inadequacy of such a test of death is patent. First, the fact that circulation will not restart spontaneously is consistent with life, as any beneficiary of cardiopulmonary resuscitation will testify. Second, the difficulty of knowing whether something will *not* happen is obvious. The usual protocol for permanent cessation is two to five minutes [45, p. 315], yet as is known from the Lazarus Phenomenon, spontaneous heartbeat can resume up to (at least) ten minutes following prolonged CPR [46]. Given this, if medical intervention will not be carried out to restart circulation, the chance of spontaneous autoresuscitation is severely diminished and the permanence test becomes, as it were, self-fulfilling. Third, why should it matter whether a function is spontaneous, as long as it can be supplied artificially? One might as well dismiss amputees as unable to walk because they cannot spontaneously regrow a limb. Why bother with a prosthesis?

Suppose, now, that irreversibility is defined as the inability to restore function with current technology. The inadequacy of such a definition stares one in the face. How could the question of whether a person is literally dead depend on what technology happens to be around for reviving them? Should one say that an apnoeic drowning victim, being in exactly the same physiological condition in a world with the technology of 1650 and in a world with the technology of 2018, would be literally dead in the former but alive in the latter? Those whose circulation cannot be restored with current technology may well be ‘as good as dead’ or on a fast descent towards death—but, depending on the circumstances, they may literally be alive.

If irreversibility is defined in terms of lack of restorability of a function with any future technology, the fact of death is held hostage to human ingenuity. To be sure, a person who is, in fact, dead is not capable of restoration to life using any future technology—only a miracle could achieve this—but they are not dead *because* of this technological impossibility. Rather, the technological impossibility itself, along with the death, is due to the laws of nature. So the question simply becomes one of when a function cannot be restored due to the laws of nature. On the hylemorphic theory, there is no simple scientific test of this for living things; rather, a metaphysical judgment has to be made concerning the integrity of the organism. Is the organism as a whole present and doing what organisms of its type essentially do?

It seems that any other definition of irreversibility will be question begging, since it will explicitly or, more likely, implicitly appeal to the very loss of integrity about which one is trying to make a determination in the first place. In other words, irreversibility in the metaphysical rather than the technological sense—something like irreversibility in principle—is a symptom rather than a criterion of death. It is because an organism is dead that nothing—whether natural, artificial, present, future, or possible—can occur or be performed to restore whatever function, including circulation, whose loss one might deem to be as close as one can get to the moment of death.

## Putrefaction as the only sure sign of death

The second problem with Shewmon’s position concerns his denial of a single, universally valid sign of death. Yet he recognises that decomposing bodies are certainly dead. I contend, given the above discussion, that only putrefaction—the physical decomposition of the body—can be a certain sign that death has occurred. Putrefaction is by far the surest sign that, as Shewmon puts it, the ‘anti-entropic processes at the basis of life’ [13, p. 466] have disappeared. Physical disintegration is, by its very nature, the manifestation of disintegration at the metaphysical level—the loss of organismic integrity that must occur if the form has been separated from matter.

Decomposition involves both autolysis—the self-digestion of the organism by its own enzymes—and heterolysis, which is the digestion of the organism by internal and external microbes and other organisms (such as necrophagous insects).^30^ The process goes through several stages, beginning with algor mortis, which is the changing of the body’s temperature (usually cooling) to match the ambient temperature, and ending with dry remains, usually teeth and bones. In between are various stages, such as rigor mortis—the stiffening of the body—and livor mortis, or lividity, which is the settling of the blood, via gravity, to the portions of the body closest to the ground.

There is, of course, not a sane observer, professional or not, who would deny that such phenomena are certain signs of death.^31^ So why should these—at least, the earliest stages such as algor, rigor, and livor mortis—not be agreed upon as the definitive test of death? Such has been the practice in most cultures since the dawn of humanity.^32^ Of rigor mortis, Shewmon says: ‘Rigor mortis is a valid criterion far from the moment of death, and therefore not a clinically very useful one’ [41, p. 250]. There are two problems with this remark. First, what does ‘far from the moment of death’ mean? Rigor mortis, depending on circumstances, can set in as early as ten minutes after death [47, p. 75]. Is that far from the moment of death?^33^ It would be earlier than many other cases in which anything close to irreversible cessation of cardio-circulatory function occurs. Second, what does ‘clinically not very useful’ mean? If it means ‘not useful for knowing whether to attempt resuscitation’, putrefaction is eminently useful. If it means ‘not useful for scientific purposes’, then a forensic pathologist would keenly disagree.

Perhaps Shewmon means ‘not useful for organ procurement’? In the mouth of many other writers on this topic, that would appear the most likely interpretation. Organs belonging to a decomposing body are of no clinical use whatsoever. I am reluctant, however, to attribute this interpretation to Shewmon, the entire thrust of whose writings on this subject runs counter to the eagerness expressed by so many clinicians and ethicists to loosen the criterion of death so as to make ‘organ harvesting’ easier and more abundant. It may be, though I am not sure, that Shewmon is implicitly endorsing circulatory death as a criterion of death for transplantation purposes. Perhaps he would want to extend the so-called ‘stand-off’ period to longer than five minutes, but he would then be running the risk of undercutting the possibility of transplantation itself, since the longer the interval, the less likely organs are to be usable. Given the possibility even with current technology of restoring both cardiac and circulatory function after long periods,^34^ it is all but impossible to know what the stand-off period should be. If I am right that decomposition is the only certain sign of death, there should be no stand-off period at all.

In any case, there can be no doubt that, as far as current technology goes, putrefaction makes organ harvesting all but impossible. But if it is the only sure sign of death, it is also highly likely that, from the beginning of the transplant era in the 1950s, every single organ donor declared dead has, in reality, been alive and has, therefore, been killed for their organs—whether intentionally or not. Put another way, it would be hard to come up with a single case where an objective clinician could, in all honesty, put his hand on his heart and declare that he is as certain of the real death of the so-called ‘dead donor’ before him as he would be of a rotting corpse.

My hypothesis would be false were it known to be the case that organs have been removed from patients in whom decomposition has, in fact, begun but has not yet been detected. After all, autolysis and heterolysis begin microscopically before being susceptible to gross observation using either the naked eye or the typical instruments of the emergency ward. It is doubtful, though, whether any surgeon has ever let a patient’s dying process get that far before beginning the process of organ extraction. Quite simply, organs need to be warm, fresh, and either functioning or capable of quick restoration of function to be usable in a recipient. In the current state of things, this is just not possible when decomposition begins.

Does this mean that organ transplantation can only ever violate the dead donor rule? Once again, given current technology, I would assert this to be the case. So, it seems, would Robert Truog, who calls the dead donor rule an ‘illusion’, sustaining the idea that a ‘bright line’ can be drawn ‘between the dying process and organ procurement’ [48, p. 318]. However, whereas Truog maintains that doctors should simply abandon the rule and ensure that patients are comfortable and do not suffer when they are killed for their organs, one could hold the reverse. Although there is no space to argue for it here, I submit that patients should, quite simply, never be killed for their organs.

## Conclusion

I have enunciated a strong thesis to the effect that, prior to decomposition, it is certain that the dead donor rule is always violated given current circumstances. But even a weaker claim is serious enough—that there is more than negligible risk that the rule is violated. It would take a whole other discussion to evaluate the moral rules surrounding the risk of intentionally causing a serious wrong. This is not, in my view, a matter in which double-effect reasoning comes into play. The organ harvester is acting intentionally: the only question is whether what he is doing is an act of intentional homicide or not. If it is objectively uncertain, how serious must the risk be before he may not act? One can at least postulate that the more serious the wrong would be, the more certain it should be that the doctor would not be committing it. Homicide is the most serious natural evil a person can intentionally bring about. I would contend, therefore, that anything other than a negligible risk of committing it is an impenetrable barrier to action.

Until such time, then, as a way of preserving organ usability in people known to be dead—in other words, bodies in whom the decomposition process has begun—is discovered, a moratorium should be imposed on all transplants from all but certainly living humans who both have made a donation voluntarily^35^ and—at least if killing with consent is still wrongful—remain certainly alive afterwards.

## Footnotes

1 This is assuming, of course, that the patient is not killed before their organs are taken.

2 Cf. the ‘higher brain’ criterion—that death is the irreversible cessation of consciousness [6], albeit this broad criterion is understood in many different ways.

3 It was, of course, the President’s Commission for the Study of Ethical Problems in Medicine [7] that gave whole brain death its canonical status and continued widespread acceptance. Its most prominent defender is James Bernat; see, e.g., [8, 9], along with the highly influential [10].

4 My own view is that even with consent, this would be a wrongful killing, but that is another matter that need not detain us here.

5 The wings have not been amputated, for instance.

6 Thanks to Michael Sherwin for making me realise that what I say about the possible non-persistence of form after separation from matter applies *pari passu* to the matter itself.

7 For more on this, see [19, ch. 7.4].

8 Henceforth, I use the terms *loss* and *separation* interchangeably.

9 The same view is expressed by David Jones [20, p. 138], whose article presents a brief but useful summary of some of the key issues I explore here.

10 I apologise for the quasi-neologism, which has a brief pedigree of much older usage in this context and captures what I intend to say far better than the ambiguous term *information*.

11 For defence of this doctrine, see [19, pp. 68–71].

12 Does my argument imply that death could *never* be identified with a physiological process, given that all processes are gradual? I think this is the case, but it still does not rule out the identification of death with a physiological event, as long as the event were truly instantaneous and entailed the immediate transition of the organism from the state of integrity to the state of non-integrity. I am not convinced that there are any genuinely instantaneous events of a purely physical (or physiological) nature. But even if there were—envisioning, perhaps, some kind of sub-atomic or quantum transition—it would then need to be shown that such an event had anything whatsoever to do with organismic integrity. The task would be difficult, to say the least. I leave such explorations to another occasion.

13 Those who highlight integrity include Shewmon [11, 13] and elsewhere [7, 10, 15–17, 22].

14 Cardiac arrest, for instance, does not entail the instantaneous cessation of all circulatory function.

15 Shewmon [13, p. 468] is, in my view, completely correct.

16 Agreeing again with Shewmon [13, p. 429], following [10].

17 Cited also by Shewmon [11, pp. 462–463].

18 Patrick Lee and Germain Grisez [15] defend the consciousness (‘radical capacity for sentience’) view, while Robert Veatch [6] defends the personhood (‘higher brain’) view.

19 See in particular [11].

20 Also see [14, pp. 63–66] for discussion.

21 Here Miller and Truog refer to a case initially described by Shewmon [26, p. 1543] and later analysed by Susan Repertinger et al. [27, p. 594].

22 Maureen Condic lists, without further explanation, sexual maturation as having ‘components’ observable in ‘cells or tissues *ex vivo*’, and ‘proportional growth’ as observable in ‘organ culture’ [23, p. 259]. As to the first, it is hard to know what she is talking about, other than perhaps the observation of, say, a tissue culture sprouting pubic hair or some such. This would not be sexual maturation of the kind seen in a paradigmatic living organism, merely one small aspect of it in one part of the body. As to the second, the growth of an organ in culture is not the *bodily* growth seen in paradigmatic living organisms. What has been found in brain-dead patients is, however impaired or reduced, of the same kind as found in paradigmatic living organisms, not tissue or organ cultures.

23 See Emily Partridge et al. [30] on the artificial gestation of lambs for four weeks.

24 For a detailed account, see [31].

25 For details, see Gary Schoenwolf et al. [32, ch. 1].

26 Contra Joshua Hoffman and Gary Rosenkrantz [33, 34].

27 See further Dick Teresi [35, pp. 77, 88, 90].

28 Data calculated from statistics of the United States Department of Health and Human Services [37, 38].

29 See, for example, [11, pp. 469, 473; 13, pp. 465–467].

30 For many details concerning the decomposition process, see [40, ch. 1].

31 Lest it be objected that a putrefying body might just be a living body with gangrene, note first that gangrene is always localised, with a line of demarcation between necrotic and living tissue: there is no ‘whole-body’ gangrene. Second, gangrene is always accompanied by inflammation and repair, which are absent from a putrefying corpse.

32 When I assert that putrefaction is the only sure sign of death, note that I am talking only about the normal or typical case, by which I mean the case where there is a body in the first place. Of course, being vaporised from the ankles up is a sure ‘sign of death’, as is the liquefaction of one’s entire head. The object is always to identify the sign of loss of integrity. Lack of a body entails lack of bodily integrity. I assume that the liquefaction of one’s entire head is a pretty reliable sign of loss of integrity, but brain disappearance is not the same as brain death, so this sort of case gives no comfort to brain death theorists. Further, given the gruesome stories that circulated during the French Revolution about headless bodies walking and bodiless heads talking (some of this may be apocryphal, but not all), one should not be so quick as to assume that the radical separation of major parts of the body is a sure sign of death either, unless there is no body to speak of. What is always required is loss of integrity—of which putrefaction is the infallible sign. (Thanks to Alex Pruss for pushing me on some of these cases.)

33 This does raise the obvious question: how can we know the time of onset of rigor mortis if we do not know with certainty the time of death in the first place? Presumably, the answer is a combination of (i) forensic scientists’ use of cardio-respiratory cessation as a proxy for the moment of death and estimations based on already-observed intervals between this and rigor mortis; and (ii) experiments performed on animals where an indubitably fatal act is performed and time calculations are made.

34 See further [45, pp. 313–314].

35 Or have, on ethically permissible grounds, had consent given on their behalf.
